# Anesthetic management of a hydrocephalus patient with inclusion body myositis

**DOI:** 10.1186/s40981-017-0129-y

**Published:** 2017-10-18

**Authors:** Daiki Takekawa, Hirotaka Kinoshita, Tomoyuki Kudo, Masato Kitayama, Tetsuya Kushikata, Kazuyoshi Hirota

**Affiliations:** 1 Department of Anesthesiology, Hirosaki University Graduate School of Medicine, 5 Zaifu-cho, Hirosaki, 036-8562 Japan; 2 Division of Surgical Center, Hirosaki University Hospital, 53 Hon-cho, Hirosaki, 036-8563 Japan

**Keywords:** Inclusion body myositis, General anesthesia, Regional anesthesia

## Abstract

Inclusion body myositis (IBM) is an inflammatory muscle disease characterized by slowly progressive muscle weakness and wasting, especially affecting proximal leg and distal arm. We report a successful anesthetic management of a 68-year-old male patient with IBM undergoing ventriculoperitoneal shunt. Anesthesia was induced and maintained with total intravenous anesthesia using propofol, remifentanil, and ketamine. The trachea was uneventfully intubated without muscle relaxants. Ultrasound-guided subcostal transversus abdominis plane block and rectus sheath block were performed with 0.25% levobupivacaine 45 ml that could provide good surgical condition without muscle relaxants. Intravenous flurbiprofen 50 mg and morphine 2 mg were used for postoperative analgesia. The patient’s emergence from general anesthesia and the postoperative course was uneventful.

## Background

Inclusion body myositis (IBM) is an inflammatory muscle disease characterized by slowly progressive muscle weakness and wasting, especially affecting proximal leg and distal arm although cardiac and respiratory muscles are rarely impaired [1]. IBM is generally observed in men and in middle to late life [1]. However, dysphagia often occurs and increases the risk of aspiration [1]. Because of the rarity, there are not many case reports regarding anesthetic management of patients with IBM, and the anesthetic management has yet to be established [2]. Thus, we report a case of successful anesthetic management in a patient with IBM undergoing ventriculoperitoneal shunt.

## Case presentation

We have obtained a written informed consent from the patient for publication of this case report.

The patient was a 68-year-old man (162 cm, 49 kg). He was diagnosed with IBM in his fifties as results of the scrutiny for slowly progressive muscle weakness and wasting of proximal legs and distal arms. He took prescribed prednisolone 10 mg per day orally. Two years ago, he underwent endovascular treatment for subarachnoid hemorrhage under local anesthesia with no sequelae. However, he visited our hospital for neurological examinations because he had head abrasion by falling down. Then, he was diagnosed with hydrocephalus by computed tomographic scanning and scheduled to undergo ventriculoperitoneal shunt under general anesthesia. His consciousness was clear. The manual muscle test (MMT) of each muscle was preoperatively evaluated, and his MMTs of deltoid, biceps, triceps, flexion of wrist, extension of wrist, palmar interossei, iliopsoas, quadriceps, hamstring, and anterior tibialis were 4/4 (right/left), 5/5, 4/4, 4/4, 4/4, 3/3, 2/2, 4/4, 3/3, and 4/4, respectively. In addition, his right grip force was 8 kg and left was 5 kg. He needed some assistance for his activities of daily living and ambulant with a walker. His respiratory function test was within the normal range. He did not have any other abnormal medical history and abnormal laboratory data including serum creatine phosphokinase (CPK) level (56 U/L, normal < 170 U/L).

On the morning of the surgery, he took his daily dose of prednisolone and intravenous famotidine 20 mg as anesthetic premedication. Anesthesia was induced by propofol 60 mg, ketamine 20 mg, and remifentanil 0.5 μg/kg/min, and maintained with propofol 4 mg/kg/h and remifentanil 0.15–0.2 μg/kg/min. Tracheal intubation was uneventful without using muscle relaxants. After the induction, ultrasound-guided subcostal transversus abdominis plane (TAP) block and rectus sheath block (RSB) were performed with 45 ml of 0.25% levobupivacaine. Intravenous hydrocortisone 100 mg was administered as perioperative steroid replacement therapy. Flurbiprofen 50 mg and morphine 2 mg were also intravenously administered for postoperative analgesia. Good surgical condition was maintained by TAP and RSB without any muscle relaxants. The duration of surgery was 1 h 45 min. The patient was emergence from general anesthesia followed by tracheal extubation without any complications. Then, the patient moved to the intensive care unit, and his postoperative course was uneventful. In addition, his condition has not been deteriorated with an oral medication of 10 mg prednisolone after the discharge from our hospital.

## Discussion

Regarding general anesthesia for muscular dystrophies, total intravenous anesthesia (TIVA) has been reported to be safer than inhalational anesthesia because of the lower risk of inducing malignant hyperthermia and rhabdomyolysis [3]. However, it has not been elucidated which anesthetic method; inhalation anesthesia or TIVA may be safer for patients with inflammatory myopathies including IBM. The congenital factors of rhabdomyolysis have been reported to be metabolic causes with failure of energy production, structural causes with muscular dystrophies and myopathies, calcium pump disorder with RYR1 gene mutations, and inflammatory causes with myositis [4]. Thus, we chose TIVA because IBM is categorized as an inflammatory myopathy. However, as Mortenson and colleagues [2] reported case series of anesthesia for IBM patients showing no adverse reactions with inhalational anesthesia, both anesthetic methods might be acceptable for IBM patients.

Patients with IBM often reveal progressive weakness and atrophy of oropharyngeal muscles and diaphragm to cause dysphagia and respiratory insufficient. Consequently, the risk of postoperative pulmonary complications including aspiration pneumonia could increase. Indeed, Nakano and colleagues reported postoperative aspiration of a patient with IBM [5]. In this case, the patient was managed without muscle relaxation but developed aspiration pneumonia on the second postoperative day, which was successfully treated with antibiotics. Therefore, we gave preanesthetic famotidine to reduce gastric acidity and volume.

It has been reported that patients with some types of neuromuscular disorders are also markedly sensitive to muscle relaxants [6]. As the sensitivity of IBM patients to muscle relaxants has not been elucidated, we avoided to use muscle relaxant in the present case. However, Mortenson and colleagues reported [2] that muscle relaxant was safely used for 16 IBM patients including 8 patients revealing dysphagia, and the postoperative course was unremarkable in all patients except patients planning postoperative mechanical ventilation. Thus, muscle relaxants might safely be used for the IBM patients.

As described above, patients with neuromuscular diseases are often sensitive to muscle relaxants to cause postoperative respiratory complications such as respiratory depression and aspiration pneumonia. In addition, residual opioids may increase the postoperative respiratory complications. As regional anesthesia including peripheral nerve blocks can markedly reduce the doses of muscle relaxants and opioids, combination of general anesthesia and regional anesthesia may be suitable for patients with muscle diseases such as IBM. Epidural block may be another possible choice for combination. However, we chose peripheral nerve blocks to avoid the possibility of hypotension due to epidural block in this case.

## Conclusions

In summary, we experienced a successful anesthetic management for a patient with IBM under TIVA in combination of regional anesthesia without muscle relaxants. However, further data accumulation must be required to establish safe anesthetic management for patients with IBM.
